# Childhood-onset Caroli’s disease as a cause of recurrent fever: A case report

**DOI:** 10.3389/fped.2022.903285

**Published:** 2022-08-04

**Authors:** Jing Sun, Sheng Wang, Biquan Chen

**Affiliations:** Department of Infectious Diseases, Anhui Provincial Children’s Hospital, Hefei, China

**Keywords:** Caroli’s disease, pediatrics, case report, intrahepatic bile duct dilatation, recurrent fever

## Abstract

Caroli’s disease is a rare congenital bile duct malformation characterized by intrahepatic bile duct dilatation. This kind of situation is seldom encountered in clinical work. We report such a case who presented to our emergency department with recurrent fever as initial symptom. According to the clinical manifestation and imaging examination, a 13-year-old boy was diagnosed with suppurative cholangitis and sepsis caused by Caroli’s disease. The symptoms were got relieved after antibiotic therapy upgraded from cephalosporins to carbapenems. After 5 months of follow-up, he did not have fever, abdominal pain or any other discomfort. We believe the present report is of medical significance since it serves as a reminder that Caroli’s disease may have atypical presentations and be masked by non-specific clinical findings. The report hopes to enlighten our pediatric colleagues by providing more knowledge on such rare congenital disease.

## Introduction

Caroli’s disease is a rare congenital biliary disease characterized by non-obstructive cystic dilatation of the intrahepatic bile ducts, which may be in a focal or multifocal manner (1). The pathogenesis of Caroli’s disease is not clear, but the familial clustering suggests that some cases are inherited in autosomal recessive fashion (2). This disease is usually noted by jaundice and/or pain in the right upper abdomen. It enjoys specific feature in imaging examinations, central spot sign, which is helpful to the diagnosis. However, the treatment strategy for Caroli’s disease is still controversial and mainly depends on the clinical features and the location of the biliary abnormalities. The following is to share the diagnosis and treatment of a case of Caroli’s disease confirmed by typical imaging with no family history or specific symptoms. We also reviewed the clinical features reported in the literatures to help clinicians diagnose earlier and facilitate the treatment and management of this disease.

## Case presentation

A 13-year-old boy presented to our emergency department with complaint of recurrent fever for more than 1 month. He was born in a non-consanguineous family without history of Caroli’s disease. The first fever occurred without obvious causes in early September 2021, and the maximum temperature reached 39.0°C accompanied by headache. He was treated with anti-infective treatment in the community clinic for 4 days. Then the temperature returned to the normal range. Two weeks later, the patient got a fever above 39.0°C again, and the intervals was gradually shortened. To seek for further treatment, he went to the people’s hospital of the local city. The temperature returned to normal after 5 days of treatment with ceftriaxone and methylprednisolone for septicemia. However, no further examination was made to determine the cause of fever. The night before coming to our hospital, the boy had a higher temperature once again. Finally, he was brought to our hospital, a provincial tertiary children’s hospital. During the course of illness, he had poor appetite and sleep. After excluding the possibility of COVID-19, he was admitted to the department of infectious disease. A physical examination was remarkable for mild tenderness in the upper abdomen, but no rebound tenderness. Besides, the liver and spleen were palpable 4.0 cm under the ribs. The laboratory results after admission were shown in Supplementary Table 1. Due to the previous medication in primary hospital, the levels of inflammatory biomarkers showed just a slight increase, while the coagulation, liver function and tumor markers were in the normal range.

**FIGURE 1:**
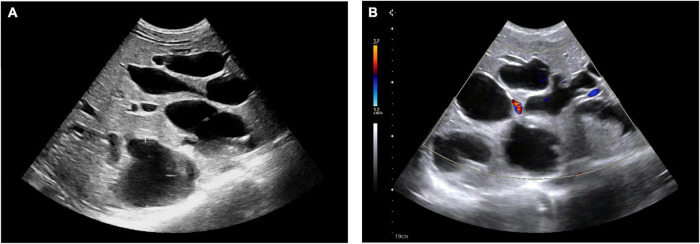
Ultrasonography showed multiple cysts in the liver **(A)**. Blood flow can be seen in few cysts **(B)**.

**FIGURE 2:**
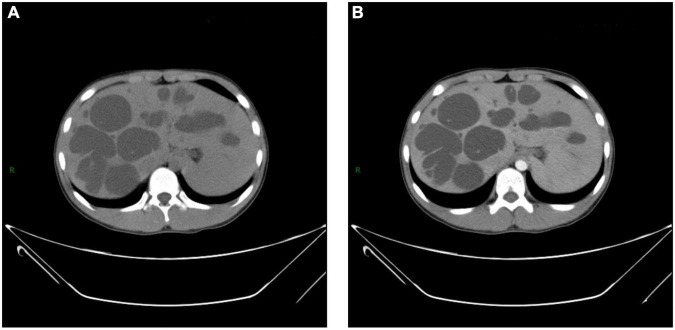
Computerized tomography (CT) shows **(A,B)** multiple hypoechoic lesions scattered in both lobes of liver. Few lesions show “central spot sign,” which is more significant in enhanced image **(B)**.

**FIGURE 3:**
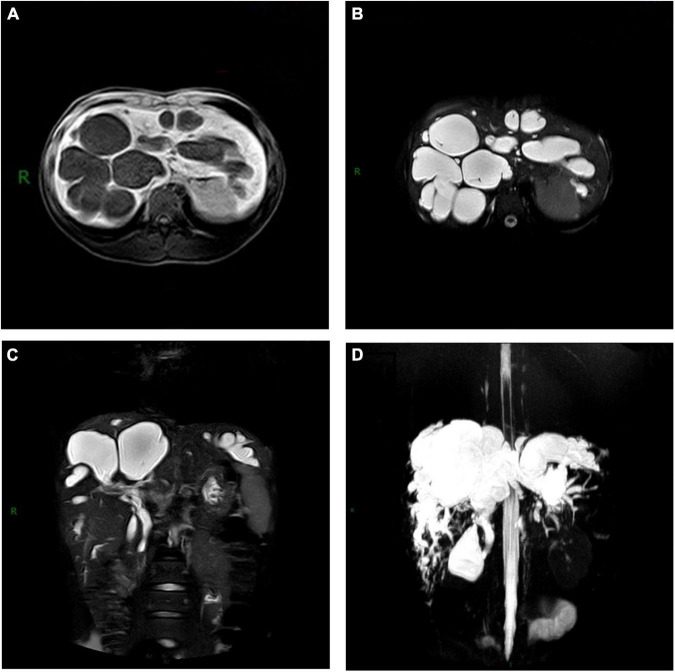
Magnetic resonance cholangiopancreatography (MRCP) of Caroli’s disease showed multiple cystic lesions of **(A)** low intensity on T1-weighted images, **(B)** high intensity on T2-weighted images and **(C,D)** connected with the biliary system.

The temperature rose to as high as 40.3°C on the 2nd day. After a comprehensive septicemia screening, empirical anti-infective treatment with cefotaxime (1.0 g q6h) was applied to the patient. 48 h later, observation on the temperature showed a constant fever over 40.0°C. Meanwhile, the tenderness in the right upper abdomen worsened. Emergency abdominal ultrasound showed multiple cysts in the liver and hepatosplenomegaly (Figure 1). At the same time, no obvious renal cyst was found. Then, an abdominal computerized tomography (CT) scan was performed and showed prominent dilation of the intrahepatic bile ducts (Figure 2). To make sure of the diagnosis, a magnetic resonance cholangiopancreatography (MRCP) was performed, which revealed multifocal dilatation of the intrahepatic bile ducts associated with hepatomegaly (Figure 3). Both CT and MRCP showed obvious “central spot sign.” Based on these imaging findings, a diagnosis of Caroli’s disease complicated with intrahepatic suppurative cholangitis was considered. We suggested genetic testing for the child and his parents, but the parents refused because of economic reasons. Repeated blood cultures were done but no positive findings. Based on the above, the antibiotic was upgraded to imipenem and cilastatin sodium (0.5 gq8h) on the 4th day. Three days later, the body temperature dropped to normal, and the abdominal pain was got relieved significantly. The patient was discharged on the 13th day and suggested to continue taking cefditoren pivoxil orally for 1 week after discharge. After 5 months of follow-up, no similar symptoms such as fever or abdominal pain recurred.

## Discussion and conclusion

We reported a unique case of Caroli’s disease in a child with recurrent fever as initial symptom, and no family history, jaundice or abdominal signs. He was clinically diagnosed as Caroli’s disease based on the “central spot sign” in CT and MRCP imaging. Considering the first onset and no severe complications, a conservative treatment and close follow-up was adopted to the present patient.

Caroli’s disease was first described by Caroli et al., which is characterized by non-obstructive segmental cystic dilatation of the intrahepatic bile ducts (3). It is extremely rare with an approximate prevalence of one in a million (4). The first onset of the symptoms mostly occurs in adolescence or early adulthood (5). Although most cases are generally diagnosed within the first two decades of life, a small number of patients may be asymptomatic for the entire life, or accidentally detected by examination at an older age (6). However, the etiology of Caroli’s disease is not yet clear. Previous researches suggest that Caroli’s disease is an autosomal recessive disease related to PKHD1 gene mutation (2). The gene is responsible for regulating fibroblasts and is widely expressed in renal tubular cells, hepatobiliary cells and other multiple organ systems (7). The mutation of PKHD1 may lead to abnormal fibrocystic protein in kidney and liver, thus Caroli’s disease is often accompanied by autosomal recessive polycystic kidney disease (ARPKD).

The clinical manifestations of Caroli’s disease are not specific, mainly including loss of appetite, weight loss, right abdominal pain, jaundice, or recurrent fever as reported in this case (5). Some patients only show chronic mild abdominal pain, or other symptoms nothing to do with the digestive system, such as cough (8). In general, septicemia caused by cholangitis is the main reason for fever. Recurrent biliary tract infection could promote the formation of intrahepatic bile duct stones, aggravate biliary obstruction, and eventually lead to biliary cirrhosis (9). The bile stasis and associated chronic cholangitis predispose patients to the development of bile duct epithelium dysplasia and cholangiocarcinoma, which is the most common hepatobiliary canceration. It is reported that the risk of cholangiocarcinoma in patients with Caroli’s disease is about 7% (10), or even higher (11, 12), which is 100 times that of the normal population.

The atypical clinical symptoms determine the difficulty of diagnosis. In the last century, the diagnosis of Caroli’s disease was rarely established prior to surgery or operative cholangiography (13). With the rapid development of examinations, the pathological morphology of the liver could be understood clearly, which brings great convenience for the diagnosis of Caroli’s disease. (i) Laboratory examination: Liver function should be paid more attention. With the recurrent attack of intrahepatic cholangitis, there may be an increase in transaminase and bilirubin, accompanied by leukocyte elevation (mainly neutrophils). As the disease continues to advance, severe hypoproteinemia may occur due to portal hypertension. (ii) Imaging examination: Ultrasonography is usually used as the first choice due to its simple and non-invasive characteristics. In the meanwhile, the kidneys can be evaluated by ultrasound to determine whether they are complicated with polycystic kidneys. CT is also an important method for early diagnosis, characterized by multiple round cystic lesions with water-like density in the liver and slightly dilated bile duct connected with the cyst. The “central spot sign” is a characteristic manifestation of Caroli’s disease, which refers to the dot-like soft tissue image in the shadow of the cysts. The pathological basis is the branches of the portal vein surrounded by the cyst wall (14). And last but not least, MRCP is of great significance in determining whether the cystic dilate is connected with the biliary system without contrast agent. Many scholars recommend that MRCP can be used as the golden standard for the diagnosis of Caroli’s disease (15). (iii) Pathological biopsy: Histologically, Caroli’s disease is characterized by fibrosis in the portal area, bile duct hyperplasia and dilatation, and bands composed of fibrous tissue and blood vessels in the cysts. Because of its invasive nature, pathological results are usually obtained after surgical treatment instead of biopsy. (iv) Gene detection: The gene detection of Caroli’s disease has not been widely carried out. As the clinical and imaging manifestations of Caroli’s disease are similar to those of primary sclerosing cholangitis (PSC), researchers have suggested two pathogenic heterozygous variants in PKHD1 gene for Caroli’s disease (16). However, due to the high cost, gene detection is recommended to patients or their family members who can afford it.

Owing to the limited cases and absence of guideline, there have been no randomized trials about the treatment of Caroli’s disease. The individual treatment strategy depends on the clinical symptoms and the extent of biliary abnormalities, including symptomatic treatment, surgical lobectomy and liver transplantation (17). For patients with mild symptoms, a conservative treatment including anti-infection, cholagogic and hepatoprotective drugs could delay the progress of the disease. But for recurrent attack of cholangitis, surgical treatment could bring a greater relief of symptoms (18). As to localized cases (limited to single lobe) in the absence of hepatic fibrosis or liver cirrhosis, hepatectomy is recommended as the best choice (19). In order to avoid reoperation caused by incomplete resection, it is recommended to use ultrasound to determine the resection line of all lesions (20). In recent years, laparoscopic surgery has gradually become a choice for patients with localized Caroli’s disease, but whether it is better than traditional laparotomy has not been clearly confirmed. For those patients with uncontrollable infection, cystic lesions involving the whole liver, severe liver fibrosis or portal hypertension, liver transplantation should be considered (21). Moreover, early evaluation of Caroli’s disease in patients with ARPKD is helpful to evaluate the feasibility of combined liver and kidney transplantation (22).

Caroli’s disease is a complex association of conditions which usually presents together with polycystic kidney lesions (6). After evaluated by imaging examinations, there is no obvious cystic lesion in the kidneys of the patient. Moreover, the child’s guardians refused genetic testing for PKHD gene (23). Of note, this case had no history of obvious abdominal pain and jaundice, which were more common symptoms in previous reports (6, 24, 25). It was quite difficult to diagnose in primary pediatric clinics, where the symptoms have been repeatedly treated and many of the diverse imaging techniques are unavailable. Fortunately, symptomatic antibiotic treatment offered a greater relief of the symptoms. However, it is frustrating that the cystic lesions have been diffusely distributed throughout the liver according to the imaging characteristics. Therefore, segmentectomy or lobectomy of the liver is not feasible and cannot be considered for our case (26). Research indicates that the overall survival rate among patients with diffuse Caroli’s disease was significantly lower than that among patients with localized Caroli’s disease (27). If the lesions worsen in the future, or combined with decompensation of portal hypertension, liver transplantation will be the only effective choice for the present patient (13).

Through the diagnosis and treatment of the case, we have summarized the following points. First, the incidence of Caroli’s disease is low, hence it is easy to escape diagnosis or be misdiagnosed. For those children with clinical symptoms such as fever of unknown origin, non-specific abdominal pain and recurrent jaundice, the possibility of Caroli’s disease should be considered. We should make full use of modern imaging technology, especially MRCP, to improve the detection rate. The treatment of Caroli’s disease is still controversial, and the prognosis of severe cases is very poor. Early detection, diagnosis and treatment can significantly improve the prognosis and reduce the incidence of complications. These are the directions that we need to work hard in the future.

## Data availability statement

The raw data supporting the conclusions of this article will be made available by the authors, without undue reservation.

## Ethics statement

Ethical review and approval was not required for the study on human participants in accordance with the local legislation and institutional requirements. Written informed consent to participate in this study was provided by the participants’ legal guardian/next of kin.

## Author contributions

JS, SW, and BC: conceptualization. JS and BC: quality assessment. JS: data collection and collation and writing – original draft. SW and BC: writing – review. All authors made a great effort to build this novel case report.

## References

[1] Desmet VJ. Congenital diseases of intrahepatic bile ducts: variations on the theme “ductal plate malformation”. *Hepatology.* (1992) 16:1069–83. 10.1002/hep.1840160434 1398487

[2] Gupta AK Gupta A Bhardwaj VK Chansoria M. Caroli’s disease. *Indian J Pediatr.* (2006) 73:233–5. 10.1007/bf02825490 16567920

[3] Caroli J Couinaud C Soupault R Porcher P Eteve J. [A new disease, undoubtedly congenital, of the bile ducts: unilobar cystic dilation of the hepatic ducts]. *Sem Hop.* (1958) 34:496–502. 13543376

[4] Jarry J Leblanc F Saric J. [Monolobar Caroli disease]. *Presse Med.* (2010) 39:847–8. 10.1016/j.lpm.2009.10.018 20202783

[5] Yonem O Bayraktar Y. Clinical characteristics of Caroli’s disease. *World J Gastroenterol.* (2007) 13:1930–3. 10.3748/wjg.v13.i13.1930 17461492 PMC4146968

[6] Zhang DY Ji ZF Shen XZ Liu HY Pan BJ Dong L. Caroli’s disease: a report of 14 patients and review of the literature. *J Dig Dis.* (2012) 13:491–5. 10.1111/j.1751-2980.2012.00619.x 22908976

[7] Gunay-Aygun M. Liver and kidney disease in ciliopathies. *Am J Med Genet C Semin Med Genet.* (2009) 151c:296–306. 10.1002/ajmg.c.30225 19876928 PMC2919058

[8] Kaszyńska A Zielonka TM Życińska K. [A cough in a patient with cholangitis in the course of Caroli’s disease. Case report]. *Wiad Lek.* (2019) 72:294–7. 30903791

[9] Tsuchida Y Sato T Sanjo K Etoh T Hata K Terawaki K Evaluation of long-term results of Caroli’s disease: 21 years’ observation of a family with autosomal “dominant” inheritance, and review of the literature. *Hepatogastroenterology.* (1995) 42:175–81. 7672768

[10] Dayton MT Longmire WP Jr. Tompkins RK. Caroli’s disease: a premalignant condition? *Am J Surg.* (1983) 145:41–8. 10.1016/0002-9610(83)90164-2 6295196

[11] Abdalla EK Forsmark CE Lauwers GY Vauthey JN. Monolobar Caroli’s disease and cholangiocarcinoma. *HPB Surg.* (1999) 11:271–6;discussion276–7. 10.1155/1999/70985 10468120 PMC2423982

[12] Bismuth H Krissat J. Choledochal cystic malignancies. *Ann Oncol.* (1999) 10(Suppl. 4):94–8. 10436795

[13] Ananthakrishnan AN Saeian K. Caroli’s disease: identification and treatment strategy. *Curr Gastroenterol Rep.* (2007) 9:151–5. 10.1007/s11894-007-0010-7 17418061

[14] Perricone G Vanzulli A. Education and imaging. Hepatology: “central dot sign” of Caroli syndrome. *J Gastroenterol Hepatol.* (2015) 30:234. 10.1111/jgh.12828 25619235

[15] Salvadori PS Torres US D’ippolito G. Contrast-enhanced magnetic resonance cholangiography with gadoxetic-acid-disodium for the detection of biliary-cyst communication in Caroli disease. *Gastroenterol Hepatol.* (2016) 39:669–70. 10.1016/j.gastrohep.2015.07.012 26596209

[16] Gu DH Park MS Jung CH Yoo YJ Cho JY Lee YH Caroli’s disease misdiagnosed as intraductal papillary neoplasm of the bile duct. *Clin Mol Hepatol.* (2015) 21:175–9. 10.3350/cmh.2015.21.2.175 26157755 PMC4493361

[17] Cabral Correia P Morgado B. Caroli’s disease as a cause of chronic epigastric abdominal pain: two case reports and a brief review of the literature. *Cureus.* (2017) 9:e1701. 10.7759/cureus.1701 29159008 PMC5690396

[18] Bockhorn M Malagó M Lang H Nadalin S Paul A Saner F The role of surgery in Caroli’s disease. *J Am Coll Surg.* (2006) 202:928–32. 10.1016/j.jamcollsurg.2006.02.021 16735207

[19] Yilmaz S Kirimlioglu H Kirimlioglu V Isik B Coban S Yildirim B Partial hepatectomy is curative for the localized type of Caroli’s disease: a case report and review of the literature. *Surgeon.* (2006) 4:101–5. 10.1016/s1479-666x(06)80039-9 16623167

[20] Zidan A Bauschke A Scheuerlein H Settmacher U Rauchfuss F. Re-resection of remnant Caroli syndrome six years after the first resection (case report). *Int J Surg Open.* (2016) 3:8–10. 10.1016/j.ijso.2016.04.004

[21] Mabrut JY Kianmanesh R Nuzzo G Castaing D Boudjema K Létoublon C Surgical management of congenital intrahepatic bile duct dilatation, Caroli’s disease and syndrome: long-term results of the French association of surgery multicenter study. *Ann Surg.* (2013) 258:713–21;discussion721. 10.1097/sla.0000000000000269 24121258

[22] Kumar A Akselrod D Prikis M. Caroli disease revisited: a case of a kidney transplant patient with autosomal polycystic kidney disease and recurrent episodes of cholangitis. *Transplant Proc.* (2019) 51:541–4. 10.1016/j.transproceed.2018.12.025 30879585

[23] Giacobbe C Di Dato F Palma D Amitrano M Iorio R Fortunato G. Rare variants in PKHD1 associated with Caroli syndrome: two case reports. *Mol Genet Genomic Med.* (2022) e1998. 10.1002/mgg3.1998 [Epub ahead of print]. 35715958 PMC9356553

[24] Mittal D Mandelia A Bajpai M Pandey RK Dash NR. Unilobar Caroli’s disease and its management in an 8-year-old girl. *J Indian Assoc Pediatr Surg.* (2013) 18:90–1. 10.4103/0971-9261.109365 23798816 PMC3687156

[25] Almohtadi A Ahmed F Mohammed F Sanhan M Ghabisha A Al-Moliki L. Caroli’s disease incidentally discovered in a 16-years-old female: a case report. *Pan Afr Med J.* (2022) 41:204. 10.11604/pamj.2022.41.204.34088 35685111 PMC9146596

[26] Narayanasamy K Mohan J Sivanesan K Babu S Kumar P Rajendran KA. Rare congenital disorder of intrahepatic bile ducts. *J Clin Exp Hepatol.* (2016) 6:65–7. 10.1016/j.jceh.2016.02.004 27194900 PMC4862099

[27] Lewin M Desterke C Guettier C Valette PJ Agostini H Franchi-Abella S Diffuse versus localized Caroli disease: a comparative MRCP study. *Am J Roentgenol.* (2021) 216:1530–8. 10.2214/ajr.20.23522 33881897

