# Supplementary material for: Childhood-onset Caroli’s disease as a cause of recurrent fever: A case report
Source: Front Pediatr. 2022 Aug 4;10:903285. doi: 10.3389/fped.2022.903285 (PMC9386290; doi:10.3389/fped.2022.903285)
Supplement: Supplementary file 1 [file Table_1.DOC]

**Table 1** Laboratory data of peripheral blood on admission.

| **Laboratory test** | Result |
| --- | --- |
| COVID-19 nucleic acid test | Negative |
| Blood cultures | Negative |
| CRP (mg/L) | 38.80↑ |
| PCT (ug/L) | 0.27↑ |
| Complete Blood Count |  |
| WBC x 109/L | 10.13↑ |
| Neutrophil (%) | 85.90↑ |
| Hb (g/dl) | 12.4 |
| PLT x 109/L | 172 |
| Coagulation function |  |
| PT (sec) | 12.30 |
| APTT (sec) | 34.90 |
| Fg (g/L) | 3.38 |
| D-Dimer (mg/L) | 0.19 |
| Liver function |  |
| TB (umol/L) | 10.4 |
| ALB (g/L) | 74.7 |
| ALT (U/L) | 21.0 |
| AST (U/L) | 16.0 |
| Tumor markers |  |
| CEA (ng/ml) | 0.0 |
| AFP (ng/ml) | 1.3 |
| HCG (mIU/mL) | <2.0 |
| SF (ng/ml) | 184.00 |
| ANA | Negative |
| ANCA | Negative |

COVID-19 = Corona Virus Disease 2019, CRP = C-reactive protein, PCT = procalcitonin, WBC = white blood cell, Hb = hemoglobin, PLT = platelet, APTT = activated partial thromboplastin time, PT = prothrombin time, Fg = fibrinogen, TB = total bilirubin, ALB = albumin, ALT = alanine aminotransferase, AST = aspartate aminotransferase, CEA = carcinoembryonic antigen, AFP = Alpha fetoprotein, HCG = human chorionic gonadotropin, SF = serum ferritin, ANA = anti-nuclear antibody, ANCA = anti-neutrophil cytoplasmic antibody.
